# Genomic and Molecular Analyses Identify Molecular Subtypes of Pancreatic Cancer Recurrence

**DOI:** 10.1053/j.gastro.2021.09.022

**Published:** 2022-01

**Authors:** Stephan B. Dreyer, Rosie Upstill-Goddard, Assya Legrini, Andrew V. Biankin, Glasgow Precision Oncology Laboratory, Sarah Allison, Andrew V. Biankin, Dario Beraldi, Euan Cameron, David K. Chang, Susanna L. Cooke, Richard Cunningham, Stephan Dreyer, Paul Grimwood, Shane Kelly, John Marshall, Brian McDade, Elizabeth A. Musgrove, Donna Ramsay, Rosie Upstill-Goddard, Lisa Evers, Selma Rebus, Lola Rahib, Bryan Serrels, Nigel B. Jamieson, Colin J. McKay, Paul Westwood, Nicola Williams, Fraser Duthie, William Shen, Antonio Pea, Amber L. Johns, Anthony J. Gill, Lorraine A. Chantrill, Paul Timpson, Angela Chou, Marina Pajic, Tanya Dwarte, David Herrmann, Claire Vennin, Thomas R. Cox, Brooke Pereira, Shona Ritchiee, Daniel A. Reed, Cecilia R. Chambers, Xanthe Metcalf, Max Nobis, Gloria Jeong, Lara Kenyon, Ruth J. Lyons, Nicola Waddell, John V. Pearson, Ann-Marie Patch, Katia Nones, Felicity Newell, Pamela Mukhopadhyay, Venkateswar Addala, Stephen Kazakoff, Oliver Holmes, Conrad Leonard, Scott Wood, Sean M. Grimmond, Oliver Hofmann, Jaswinder S. Samra, Nick Pavlakis, Jennifer Arena, Hilda A. High, Ray Asghari, Neil D. Merrett, Amitabha Das, Peter H. Cosman, Kasim Ismail, Alina Stoita, David Williams, Allan Spigellman, Duncan McLeod, Judy Kirk, James G. Kench, Peter Grimison, Charbel Sandroussi, Annabel Goodwin, R. Scott Mead, Katherine Tucker, Lesley Andrews, Michael Texler, Cindy Forrest, Mo Ballal, David Fletcher, Maria Beilin, Kynan Feeney, Krishna Epari, Sanjay Mukhedkar, Nikolajs Zeps, Nan Q. Nguyen, Andrew R. Ruszkiewicz, Chris Worthley, John Chen, Mark E. Brooke-Smith, Virginia Papangelis, Andrew D. Clouston, Andrew P. Barbour, Thomas J. O’Rourke, Jonathan W. Fawcett, Kellee Slater, Michael Hatzifotis, Peter Hodgkinson, Mehrdad Nikfarjam, James R. Eshleman, Ralph H. Hruban, Christopher L. Wolfgang, Aldo Scarpa, Rita T. Lawlor, Vincenzo Corbo, Claudio Bassi, Andrew V. Biankin, Nigel B. Jamieson, David K. Chang, Stephan B. Dreyer, Nigel B. Jamieson, David K. Chang, Australian Pancreatic Genome Initiative, Nigel B. Jamieson, David K. Chang

**Affiliations:** Wolfson Wohl Cancer Research Centre, Institute of Cancer Sciences, University of Glasgow, Bearsden, Glasgow, Scotland, United Kingdom; West of Scotland Pancreatic Unit, Glasgow Royal Infirmary, Glasgow, Scotland, United Kingdom; Wolfson Wohl Cancer Research Centre, Institute of Cancer Sciences, University of Glasgow, Bearsden, Glasgow, Scotland, United Kingdom; Wolfson Wohl Cancer Research Centre, Institute of Cancer Sciences, University of Glasgow, Bearsden, Glasgow, Scotland, United Kingdom; Wolfson Wohl Cancer Research Centre, Institute of Cancer Sciences, University of Glasgow, Bearsden, Glasgow, Scotland, United Kingdom *and* West of Scotland Pancreatic Unit, Glasgow Royal Infirmary, Glasgow, Scotland, United Kingdom; 1 Glasgow Precision Oncology Laboratory, University of Glasgow, Institute of Cancer Sciences, Wolfson Wohl Cancer Research Centre, Glasgow, Scotland, United Kingdom; 2 West of Scotland Pancreatic Unit, Glasgow Royal Infirmary, Glasgow, Scotland, United Kingdom; 3 Department of Pathology, Southern General Hospital, Greater Glasgow & Clyde NHS, Glasgow, Scotland, United Kingdom; 4 West of Scotland Genetic Services, NHS Greater Glasgow and Clyde, Queen Elizabeth University Hospital Campus, Glasgow, Scotland, United Kingdom; 1 The Kinghorn Cancer Centre, Garvan Institute of Medical Research, Darlinghurst, Sydney, New South Wales, Australia; 2 QIMR Berghofer Medical Research Institute, Herston, Queensland, Australia; 3 University of Melbourne, Centre for Cancer Research, Victorian Comprehensive Cancer Centre, Melbourne, Victoria, Australia; 4 Institute for Molecular Bioscience, University of Queensland, St Lucia, Queensland, Australia; 5 Royal North Shore Hospital, St Leonards, New South Wales, Australia; 6 Bankstown Hospital, Bankstown, New South Wales, Australia; 7 Liverpool Hospital, Liverpool, New South Wales, Australia; 8 St Vincent’s Hospital, Darlinghurst, New South Wales, Australia; 9 Westmead Hospital, Westmead, New South Wales, Australia; 10 Royal Prince Alfred Hospital, Camperdown, New South Wales, Australia; 11 Prince of Wales Hospital, Randwick, New South Wales, Australia; 12 Fremantle Hospital, Fremantle, Western Australia, Australia; 13 St John of God Healthcare, Subiaco, Western Australia, Australia; 14 Royal Adelaide Hospital, Adelaide, South Australia, Australia; 15 Flinders Medical Centre, Bedford Park, South Australia, Australia; 16 Envoi Pathology, Herston, Queensland, Australia; 17 Princess Alexandra Hospital, Woolloongabba, Queensland, Australia; 18 Austin Hospital, Heidelberg, Victoria, Australia; 19 Johns Hopkins Medical Institute, Baltimore, Maryland, USA; 20 ARC-NET Center for Applied Research on Cancer, University of Verona, Verona, Province of Verona, Italy; 21 Wolfson Wohl Cancer Research Centre, Institute of Cancer Sciences, University of Glasgow, Bearsden, Glasgow, Scotland, United Kingdom; 22 Wollongong Hospital, Illawarra and Shoalhaven Local Health District, Wollongong, New South Wales, Australia; 23 Epworth HealthCare, Richmond, Victoria, Australia; Wolfson Wohl Cancer Research Centre, Institute of Cancer Sciences, University of Glasgow, Bearsden, Glasgow, Scotland, United Kingdom; West of Scotland Pancreatic Unit, Glasgow Royal Infirmary, Glasgow, Scotland, United Kingdom; Wolfson Wohl Cancer Research Centre, Institute of Cancer Sciences, University of Glasgow, Bearsden, Glasgow, Scotland, United Kingdom; West of Scotland Pancreatic Unit, Glasgow Royal Infirmary, Glasgow, Scotland, United Kingdom; Wolfson Wohl Cancer Research Centre, Institute of Cancer Sciences, University of Glasgow, Bearsden, Glasgow, Scotland, United Kingdom *and* West of Scotland Pancreatic Unit, Glasgow Royal Infirmary, Glasgow, Scotland, United Kingdom; Wolfson Wohl Cancer Research Centre, Institute of Cancer Sciences, University of Glasgow, Bearsden, Glasgow, Scotland, United Kingdom *and* West of Scotland Pancreatic Unit, Glasgow Royal Infirmary, Glasgow, Scotland, United Kingdom

Pancreatic cancer (PC) remains a highly lethal malignancy, and most patients with localized disease that undergo surgical resection still succumb to recurrent disease. Pattern of recurrence after pancreatectomy is heterogenous, with some studies illustrating that site of recurrence can be associated with prognosis.^1^ Another study suggested that tumors that develop local and distant recurrence can be regarded as a homogenous disease with similar outcomes.^2^ Here we investigate novel molecular determinants of recurrence pattern after pancreatectomy for PC.

Recurrence patterns were classified as liver, lung, local only, and other distant, whereas the no recurrence group was defined as those that did not develop any recurrence during the study period (minimum of 24 months of follow-up).^3^ Genomic, transcriptomic, immunohistochemical, and clinical data of primary resected tumor specimens from the Australian Pancreatic Cancer Genome Initiative (Australian contribution to the International Cancer Genome Consortium) PC cohort were used in the molecular analysis (n = 435). Full methods and description of cohort undergoing each analysis are available in the Supplementary Methods section.

Liver metastases after pancreatectomy (median survival, 16.0 months) was associated with significantly worse disease-specific survival than lung (29.7 months) and local recurrence (25.6 months; *P* < .001; Supplementary Figure 1*a*). Liver recurrence after pancreatectomy was associated with poor tumor differentiation (*P* < .001). Margin status (*P* = .217) and lymph node status did not predict recurrence patterns (*P* = .062).

Patients from the Australian Pancreatic Cancer Genome Initiative cohort were categorized based on transcriptional expression of the primary tumor as squamous (basal-like) or classical.^4^ Squamous tumors correlated with liver recurrence and short disease-free survival after pancreatectomy (*P* < .001; Figure 1*a*, Supplementary Figure 1*b*). Conversely, lung recurrence significantly correlated with the classical subtype (*P* = .007).

**Figure 1:**
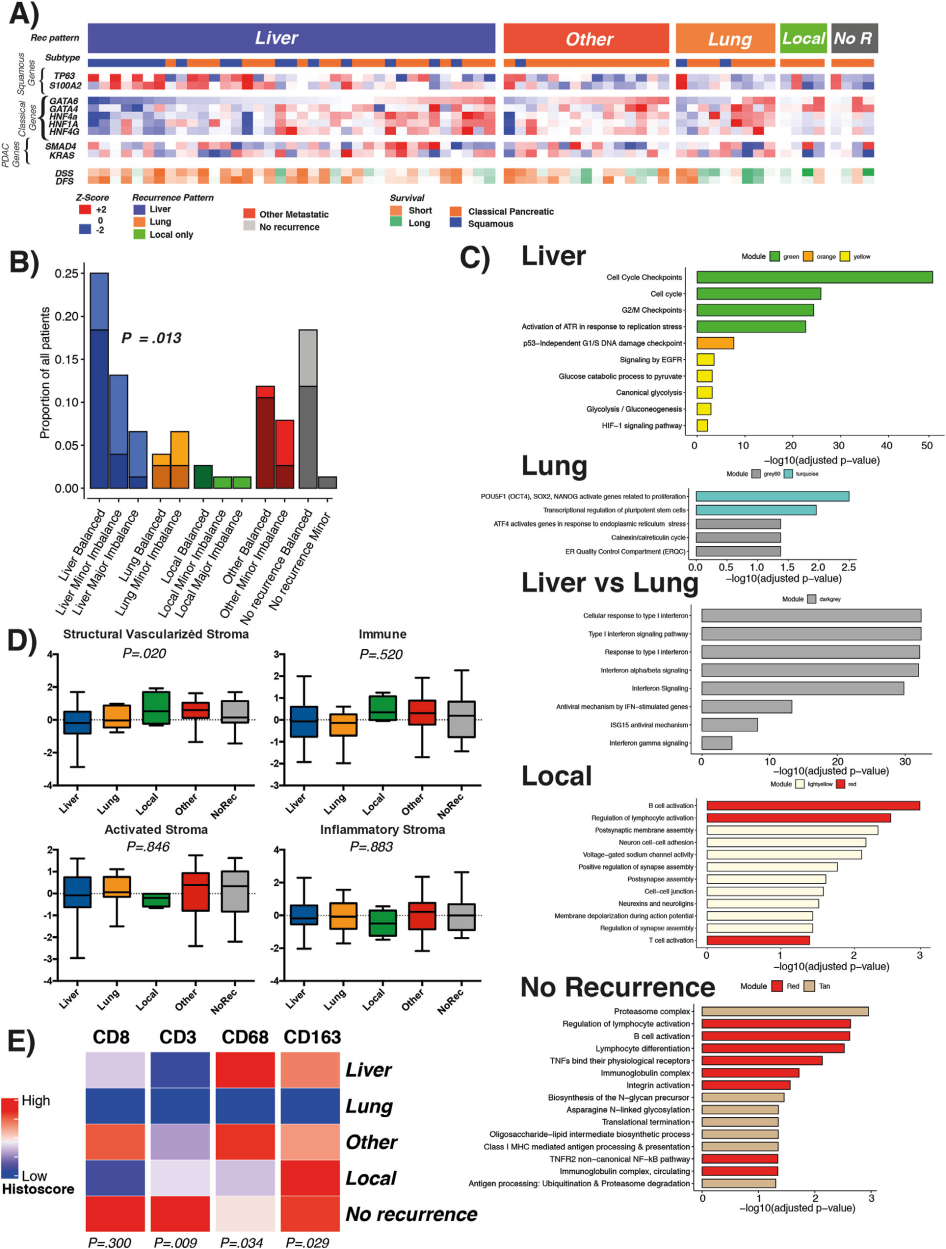
(*A*) Heatmap of RNA sequenced cases with disease recurrence pattern. Heatmap demonstrates recurrence pattern, molecular subtype, relative expression of key genes of PC, classical and squamous subtype lineage, and outcome. (*B*) *KRAS* allelic imbalance and whole genome doubling in recurrence patterns. Scale is based on proportion of overall cohort. *Dark shade* represents no whole genome doubling and *light shade* whole genome doubling for each recurrence pattern stratified by *KRAS* allelic status. *P* value calculated for *KRAS* imbalance in liver versus all other recurrence patterns using chi-square test. (*C*) Relevant molecular pathways enriched in specific recurrence patterns categorized by gene module defined by Bailey et al.^4^ Size of bar proportional to statistical weight, horizontal scale numerical for *P*. (*D*) Stromal signatures as defined by Puleo et al^7^ for each recurrence pattern. *P* calculated as analysis of variance between groups. (*E*) Heatmap of relative immune cell infiltration histoscore in different recurrence patterns. Histoscore represents cumulative score for all tissue microarray cores per patient.

The proportion of *BRAF* and *RNF43* mutations were higher in the liver and no recurrence groups, respectively (Supplementary Figure 1*c*). These failed to be below the *P* < .05 significance level, likely due to insufficient power due to the infrequent mutations. Significantly mutated gene analyses identified *RNF43* mutations (*q* < 0.001) to be associated with the no recurrence group and *BRAF* mutations (*q* = 0.020) to be associated with the liver recurrence group (Supplementary Figure 1*d*). No other gene mutation was significantly associated with a recurrence pattern. Only 1 *RNF43* mutant was pathologically described as an intraductal papillary mucinous neoplasm (IPMN) with invasion (Supplementary Figure 1*e*). In those that developed no recurrence, almost all patients (90%) had balanced *KRAS* allelic status^5^ (Supplementary Figure 2*a*), whereas *KRAS* imbalance was a feature of primary tumors that developed liver recurrence (Figure 1*b*, Supplementary Figure 2*a*). *KRAS* wild type or mutation type did not associate with any recurrence pattern (Supplementary Figure 2*a*).

Transcriptional networks and gene programs that were identified as key features of the squamous subtype^4^^,^^6^ significantly associated with liver recurrence (Figure 1*a*, Supplementary Figure 2*b*). Pathways that predispose to liver recurrence included cell cycle checkpoint activation, epidermal growth factor signaling, glycolysis, and hypoxia (Figure 1*c*, Supplementary Figure 2*c*).

Lung recurrence was strongly associated with the classical subtype and was enriched for transcriptional pathways regulating pluripotent stem cells and endoplasmic reticulum stress (Figure 1*c*).

Next, we investigated additional molecular differences between liver and lung recurrence, the most common metastatic sites in PC. In addition to enrichment in the gene programs described, liver recurrence was, relative to lung recurrence, enriched in pathways associated with innate immune response, interferon signaling, and antiviral response (Figure 1*c*).

Local recurrence was enriched for networks associated with neuronal signaling and neuron cell-cell interaction (Figure 1*c*). This may reflect the local infiltrative nature of these tumors into the peri-pancreatic nerve plexuses which predisposes to local recurrence despite negative resection margins.

Local and no recurrence groups were associated with transcriptional networks associated with immunogenic activation and infiltration (Figure 1*c*). By applying the stromal subtypes described by Puleo et al,^7^ enrichment of the structural vascularized stroma subtype (*P* = .020) was found in the local recurrence group *(*Figure 1*d*). Immunohistochemistry of the International Cancer Genome Consortium (ICGC) cohort showed that those that developed no recurrence were enriched for infiltration of CD3+ T cells (*P* = .009), and the local and no recurrence groups were enriched for CD163+ macrophages (*P* = .029; Figure 1*e*). The liver and other recurrence groups were relatively enriched for CD68+ macrophages (*P* = .034).

Here we demonstrate the impact of novel, previously undefined molecular features of the primary tumor on spatiotemporal recurrence patterns after pancreatectomy for PC. Liver recurrence is associated with significantly shorter disease-free and overall survival. *TP63* expression, cell cycle checkpoint activation, epidermal growth factor signaling, glycolysis, and hypoxia are features of the squamous (basal-like) subtype and strongly associated with liver recurrence. When compared with lung recurrence, the liver recurrence group was enriched for inflammatory pathways likely driven by chronic inflammation secondary to genomic instability and constitutional STING (Stimulator of Interferon Genes) activation that is a feature of the squamous subtype.^8^

Lung recurrence was associated with the classical subtype and longer disease-free and overall survival. In addition, local and no recurrence groups had very similar transcriptomic profiles with favorable immune signaling compared with those that develop distant metastases based on transcriptome. The no recurrence group was enriched for CD3+ immune cell infiltration. Quantification of immune cell infiltration alone may be insufficient to delineate specific stromal signaling and its influence of this on recurrence pattern and highlights the potential contribution of a cell autonomous signaling mechanism. The no recurrence group was enriched for *RNF43* mutations, yet only 1 (lung recurrence group) of the *RNF43* mutants histologically resembled IPMN with invasion and this does not explain the better prognosis associated with these mutations. This highlights the association of these mutations with primary PC out with the setting of transformed IPMN.

This study is limited by only having primary tumor samples available for analysis. Parallel molecular profiling of primary and metastatic tumors, accrued through multi-institutional studies, such as Precision-Panc, will allow more detailed analyses of pathways and processes that define and promote recurrent disease.

In summary, our results demonstrate that resected pancreatic cancers should not be considered to harbor the same “systemic” disease. Liver recurrence is the dominant spatiotemporal phenotype, with primary tumors enriched for specific molecular features that differ from those that develop lung or local recurrence. Delineating these processes, and their influence on priming the metastatic niche, dissemination, and seeding of tumor cells warrants further study to inform personalized adjuvant antimetastatic agents and surveillance strategies.

## Figures and Tables

**Supplementary Figure 1:**
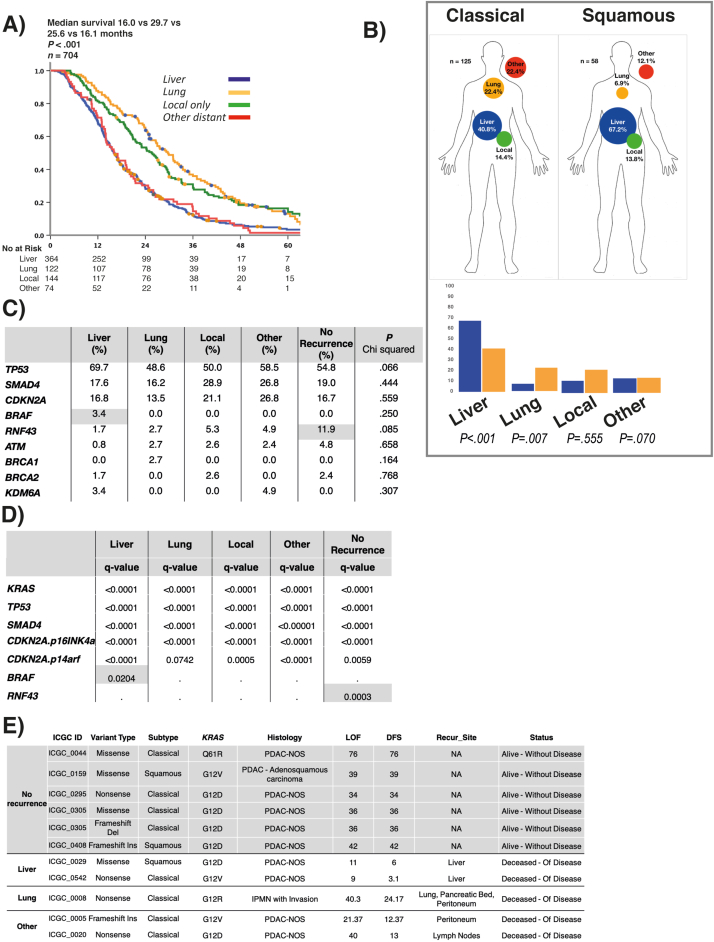
(*A*) Kaplan-Meier curve of disease-specific survival stratified by recurrence pattern in all 3 cohorts (total n = 1087). Only those with recurrent disease (n = 704) are shown. (*B*) Proportion of each recurrence pattern in patients with classical and squamous subtype. Squamous subtype enriched for liver recurrence (*P* < .001); classical subtype associated with lung recurrence (*P* = .007). Bar chart demonstrates the relative proportions of each molecular subtype in different recurrence patterns. Proportion is based on the frequency of each recurrence pattern per molecular subtype. (*C*) Proportion of gene mutations in each recurrence pattern. *P* value calculated using chi-square. (*D*) Table of significantly mutated gene analysis in recurrence patterns. A significant q value (<0.05) represents an association between mutation and recurrence pattern. Only *BRAF* (liver) and *RNF43* (no recurrence) were significant in specific recurrence patterns. (*E*) Table of clinical, molecular, and pathologic features of *RNF43* mutants in analyzed cohort.

**Supplementary Figure 2:**
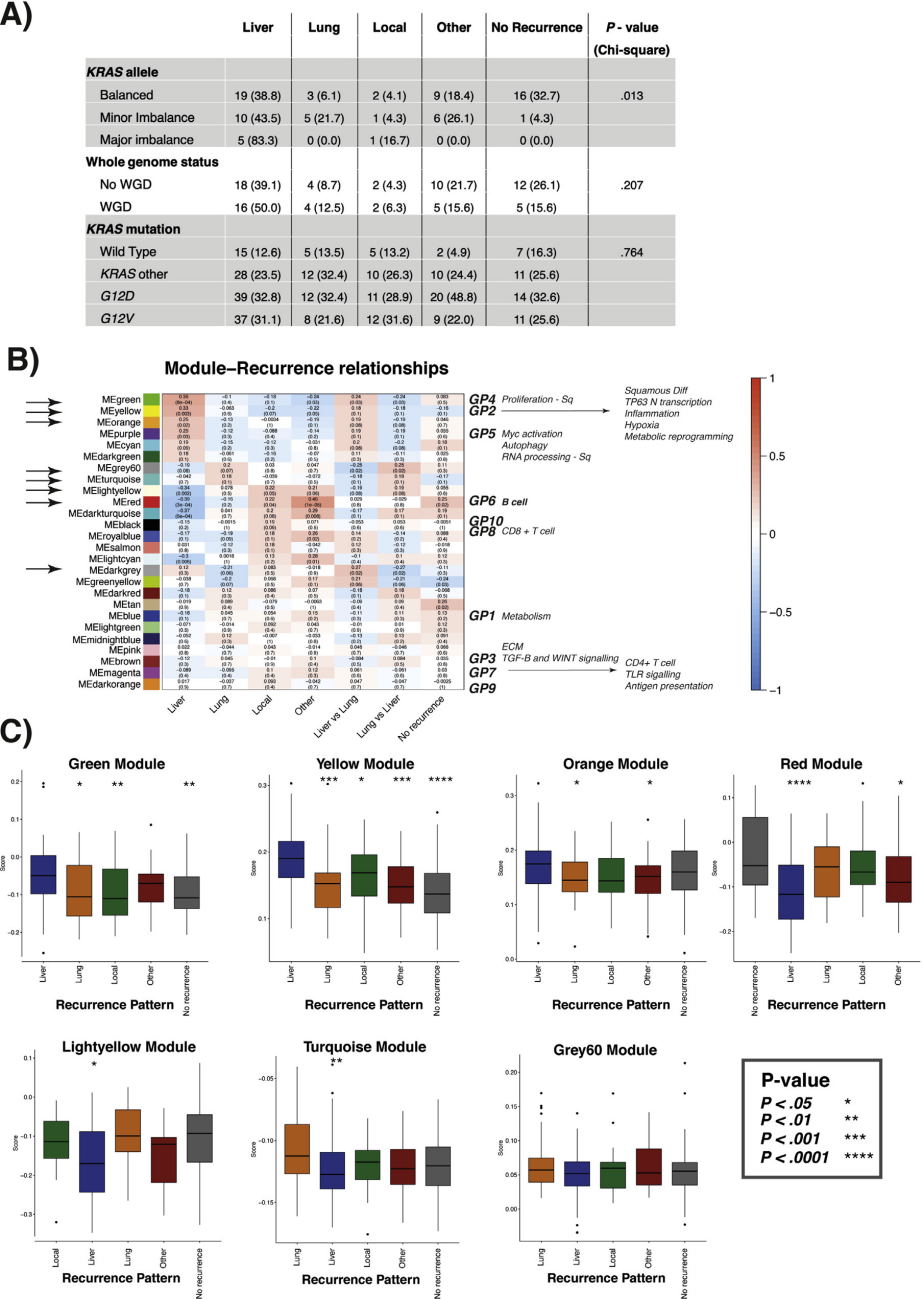
(*A*) Table demonstrating frequency of *KRAS* allelic imbalance and whole genome doubling in recurrence patterns of PCAWG cohort. (*B*) Transcriptional modules defined as significant by Bailey et al^4^ in each recurrence group for patients with RNA sequencing data. Significant modules that are associated with recurrence patterns are highlighted with *black arrow* on left y-axis. Key processes and gene programs previously described by Bailey et al^4^ that define squamous and classical pancreatic (pancreatic progenitor, immunogenic, ADEX) subtypes are highlighted along right-hand y-axis. (*C*) Relative enrichment score of genes associated with recurrence pattern in each module that associated with recurrence pattern, from the APGI microarray cohort. Statistical significance levels are ∗≤.05, ∗∗≤.01, ∗∗∗≤.001, and ∗∗∗∗≤.0001 with the first given recurrence pattern as reference (for example, for *green*, *yellow*, and *orange* modules the reference is liver, for *light yellow* it is local, and for *turquoise* and *grey 60* the reference is lung).

## Supplementary Methods

### Patient Cohort Description

Clinicopathologic and complete outcome data were obtained from prospectively maintained independent cohorts of patients with PC that underwent attempted curative resection. Patients were accrued prospectively for the Australian Pancreatic Cancer Genome Initiative (APGI) cohort (n = 437) (www.pancreaticcancer.net.au) as part of the International Cancer Genome Consortium (ICGC; www.icgc.org). Additional clinical cohorts were recruited from the West of Scotland Pancreatic Unit, Glasgow Royal Infirmary, United Kingdom (n = 367) and the Royal North Shore Hospital, Sydney, Australia (n = 283). Patients with resected oligometastatic disease were excluded. Seven hundred four developed recurrence in the study period. All patients were meticulously followed up. Patients with molecular data were followed up as per APGI protocol in keeping with standards of a prospective clinical trial. This included regular medical case notes review and contact with primary care providers and patients.

In this study, 324 had molecular and recurrence data. All molecular analyses were performed on tissue from primary resected tumors. Those with transcriptomic profiling included 96 who underwent RNA sequencing and 179 who underwent microarray gene expression. Also, 278 had both genomic (mutational) and recurrence data for analysis of mutation frequency in each recurrence pattern, whereas 78 had whole genome and recurrence data and underwent *KRAS* allelic imbalance and whole-genome doubling analysis as part of the PCAWG (Pan-Cancer Analysis of Whole Genomes) project.

Recurrence pattern were defined as the dominant recurrence phenotype: (1) liver (liver metastases along with any other sites), (2) lung (lung metastases and other sites but not liver metastases), (3) local only (only local recurrence in pancreatic bed, not lymph nodes or peritoneal metastases), and (4) other sites (this includes all other sites including peritoneal, brain, bone, and disseminated lymph nodes). No recurrence group were patients that had at least 24 months follow-up with no evidence of disease recurrence at any stage.

Ethical approval for the acquisition of data and biological material was obtained from the Human Research Ethics Committee at each participating institution.

### Statistical Analysis

Categorical variables were compared using the c^2^ test. Patients alive at the time of follow-up point were censored. The last follow-up period for patients still alive was October 2020. Kaplan-Meier survival analysis and log-rank rest were used to analyze disease-specific survival.

Fine Grays analysis was used to investigate the cumulative incidence of recurrence in different subtypes. Recurrence type was classed as the event of interest, and time was classed as disease-free-survival. Events with *P* < .05 were classed as significant. Smoothened hazard plots were then generated with patterns of recurrence and subtype plotted against disease-free survival. Smoothing was estimated from B splines assuming a Poisson distribution. Hazard ratios, *P* values, and confidence intervals were produced using univariate cox regression analysis on the hazard plots. Analysis was carried out using SPSS (Version 25.0; IBM SPSS Statistics, IBM Corporation, Armonk, NY) and R 3.6.3 (The R Project for Statistical Computing, Vienna, Austria) using packages cmprsk, bshazard, and complex heatmap.

#### Nucleic acid extraction

DNA and RNA extraction were performed using previously published methods.^1^

#### Whole-genome library preparation

Whole-genome libraries were generated using either the Illumina TruSeq DNA LT sample preparation kit (part no. FC-121–2001 and FC-121–2001; Illumina, San Diego, CA) or the Illumina TruSeq DNA PCR-free LT sample preparation kit (part no. FC-121–3001 and FC-121–3002; Illumina, San Diego, CA).

#### RNA sequencing library generation and sequencing

RNA-seq libraries were generated using TruSeq Stranded Total RNA (part no. 15031048 Rev. D April 2013) kits, used on a Perkin Elmer’s Sciclone G3 NGS Workstation (product no. SG3- 31020-0300; Perkin Elmer, Waltham, MA).

#### Library sequencing

All libraries were sequenced using the Illumina HiSeq 2000/2500 system with TruSeq SBS Kit v3 - HS (200-cycles) reagents (part no. FC-401-3001; Illumina, San Diego, CA), to generate paired-end 101 bp reads.

#### Identification of and verification of point mutations

Substitutions and indels were called using a consensus calling approach that included qSNP, GATK, and Pindel. The details of call integration, filtering and verification were done using orthogonal sequencing and matched sample approaches.

### RNAseq Analysis

ICGC cohort sequencing reads were mapped to transcripts corresponding to ensemble 70 annotations using RSEM.^2^ RSEM data were normalized using TMM implemented in “edgeR,”^3^ converted to counts per million and log_2_ transformed as previously described.^1^ Genes without at least 1 cpm in 20% of the sample were excluded from further analysis.

### Transcriptomic Profiling

The molecular subtyping criteria was generated as part of the ICGC landmark study of PC.^1^ Individual tumors were classified as either squamous or classical pancreatic subtypes. The classical pancreatic subtype encompassed the pancreatic progenitor, ADEX (aberrantly differentiated endocrine exocrine), and immunogenic subclasses described by Bailey et al.^1^

### Module Derivation and Association With Recurrence Pattern

Gene modules were derived from a transcriptional network generated from the normalized and transformed ICGC expression data as previously described.^1^ The weighted gene co-expression network analysis (WGCNA)^4^ workflow was used to generate the network and define 26 modules of coexpressed genes that were assigned colors as names.^1^ Module eigengenes were calculated and can be used as a measure of module expression in a given sample. Sample traits can be correlated to the module eigengenes to identify modules associated with those traits.

Module eigengenes for the previously defined gene modules^1^ were correlated with recurrence pattern to identify modules associated with recurrence. Lung, liver, local, and other patterns were compared to each other in a one versus rest comparison. No recurrence was compared to all other types of recurrence. Only samples with reported recurrence data were considered.

Gene enrichment analysis for significantly associated modules was performed using the Bioconductor/R package “clusterProfiler.”^5^ Module genes significantly associated with each recurrence pattern were selected for enrichment analysis using WGCNA’s “networkScreening” and filtering for genes with “q.Weighted” ≤ 0.05 and “cor.Weighted” > 0. Module association with recurrence type in the microarray samples was achieved by calculating module gene set enrichment scores using the same module genes as used in enrichment analysis. Enrichment scores for each sample were calculated using the package “singscore.”

### Puleo Stromal Component Classification

Gene set enrichment scores were calculated (“singscore”) for each sample using the 300 unique genes with highest Pearson correlation coefficient for each stromal component as previously described.^6^

### KRAS Copy Number and Whole-Genome Doubling

Copy number calls and whole-genome doubling data for samples included in the PCAWG project were obtained from the ICGC (n = 96). Samples were defined as: (1) *KRAS* balanced when major allele and minor allele counts were equal; (2) *KRAS* minor imbalance when the number of major alleles exceeded the number of minor alleles by 1 or 2; and (3) *KRAS* major imbalance when the number of major alleles exceeded the number of minor alleles by 3 or more.^7^

### Significantly Mutated Gene Analysis

The “dNdScv”^8^ was used to detect cancer driver genes (genes under positive selection) within each recurrence pattern. Driver genes are identified by using the normalized ratio of nonsynonymous to synonymous mutations (dN/dS) within the gene, where synonymous mutation rate can be taken as a proxy for expected mutation density. A high dN/dS ratio for a gene indicates that there are many more nonsynonymous mutations than expected and many of these will be genuine driver events. Mutation data for ICGC/APGI samples from the latest ICGC DCC release (26/11/2019) for which recurrence pattern information data existed were used. Results for gene-level dN/dS ratios for missense, nonsense, and essential splice mutations were obtained for all genes. Genes were reported if they had a global q value < 0.1 (q value that integrates missense, nonsense, and essential splice mutations). Genes identified as significant typically exhibit very high dN/dS ratios, indicating that they are potential driver genes.

### PC Gene Mutation Frequency

Mutation frequency of known PC-associated genes was calculated within each recurrence type. Genes were considered to be mutated in a patient if they harbored a variant of type: disruptive inframe deletion/insertion, frameshift variant, missense variant, or stop gained. Mutation data for ICGC PACA-AU (release 28) were downloaded from ICGC and were used to determine mutation frequency. Pearson chi-square tests were performed in R on the mutation frequency of each gene.

### Immunohistochemistry

Immunohistochemistry was carried out on patients (n = 213) using standard immunohistochemistry methods with markers CD3 (Vector Laboratories, Burlingame, CA), CD8 (DAKO Omnis, Agilent, Santa Clara, CA), CD68 (DAKO Omnis, Agilent, Santa Clara, CA), and CD163 (Leica Biosystems Ltd, Milton Keynes, United Kingdom). Images were analysed using the digital imaging platform HALO (Indica Labs, Albuquerque, NM). Specific analysis algorithms were developed for individual markers using an optimization tissue microarray. Briefly, the algorithm ranks Immune markers according to cellular staining intensity, giving intensity scores ranging from 0 (no positive stain) to 3 (high expression stain). Each score was then multiplied by the percentage of positively stained cells of the respective intensity score and added together. This semiquantitative measure is referred to as a Histo (H) score. A H score was generated for individual cores (3 per patient) and the overall score was calculated. Heatmaps were generated by plotting H score against recurrence type using Bioconductor/R package “ComplexHeatmap.”^9^
